# Epigenetic therapy potential of suberoylanilide hydroxamic acid on invasive human non-small cell lung cancer cells

**DOI:** 10.18632/oncotarget.11967

**Published:** 2016-09-12

**Authors:** Shirong Zhang, Kan Wu, Jianguo Feng, Zhibing Wu, Qinghua Deng, Chao Guo, Bing Xia, Jing Zhang, Haixiu Huang, Lucheng Zhu, Ke Zhang, Binghui Shen, Xufeng Chen, Shenglin Ma

**Affiliations:** ^1^ Department of Oncology, Hangzhou First People's Hospital, Nanjing Medical University, Hangzhou, China; ^2^ Department of Oncology, Affiliated Hangzhou First People's Hospital of Zhejiang Chinese Medical University, Hangzhou, China; ^3^ Department of Oncology, Hangzhou Cancer Hospital, Hangzhou, China; ^4^ Cancer Research institute, Zhejiang Cancer Hospital, Hangzhou, China; ^5^ Department of Cancer Genetics and Epigenetics, City of Hope National Medical Center, Duarte, CA, USA; ^6^ Department of Pathology and Laboratory Medicine, University of California at Los Angeles, Los Angeles, CA, USA

**Keywords:** non-small cell lung cancer, invasion, epigenetics, suberoylanilide hydroxamic acid, epigenetic therapy

## Abstract

Metastasis is the reason for most cancer death, and a crucial primary step for cancer metastasis is invasion of the surrounding tissue, which may be initiated by some rare tumor cells that escape the heterogeneous primary tumor. In this study, we isolated invasive subpopulations of cancer cells from human non-small cell lung cancer (NSCLC) H460 and H1299 cell lines, and determined the gene expression profiles and the responses of these invasive cancer cells to treatments of ionizing radiation and chemotherapeutic agents. The subpopulation of highly invasive NSCLC cells showed epigenetic signatures of epithelial-mesenchymal transition, cancer cell stemness, increased DNA damage repair and cell survival signaling. We also investigated the epigenetic therapy potential of suberoylanilide hydroxamic acid (SAHA) on invasive cancer cells, and found that SAHA suppresses cancer cell invasiveness and sensitizes cancer cells to treatments of IR and chemotherapeutic agents. Our results provide guidelines for identification of metastatic predictors and for clinical management of NSCLC. This study also suggests a beneficial clinical potential of SAHA as a chemotherapeutic agent for NSCLC patients.

## INTRODUCTION

Lung cancer is the most common cancer and the leading cause of cancer death. Despite tremendous progresses in diagnosis and treatment of lung cancer, the overall treatment outcomes remain poor. Tumor aggressiveness in metastatic lesions is the cause of lethality in lung cancer patients, and is responsible for more than 90% of failure for lung cancer treatment [1, 2].

Metastatic potential is a common feature of lung cancer. In general, lung cancer patients are often diagnosed at late stages, when the cancer has invaded local sites and produced distant metastases [3]. Development of metastasis after initial surgery is also a clinical challenge for NSCLC (non-small cell lung cancer) patients with early stage cancers. To improve the clinical management of lung cancer patients, novel strategies for diagnosis at an earlier stages and therapeutic interventions to prevent metastatic spreading of lung cancer are urgently required.

Distant metastases occur as multiple-step events: tumor cells invade into neighboring tissues and the basement membrane, intravasate into the blood stream or lymphatic flow, circulate to distant organs, and extravasate into target tissues to settle and re-grow at the new site [4, 5]. It is still not clear whether all tumor cells, or only a special subgroup of cells, have metastatic capabilities. However, there is accumulating evidence to support the latter hypothesis [6, 7]. Following the “seed and soil” theory, some rare tumor cells that escape the heterogeneous primary tumor may initiate the complicated metastatic process [8]. It is therefore of great interest, and of potential therapeutic importance, to characterize these “seed” cancer cells for a comprehensive understanding how cancer cells initiate metastatic dissemination.

In this study, we used Boyden-type cell invasion chambers coated with basement membrane extract (BME) to isolate invasive cancer cells from H460 and H1299 cell lines, and characterized the epigenetic and biological features of these cell populations. We also investigated the potential therapeutic effects of the histone deacetylase (HDAC) inhibitor suberoylanilide hydroxamic acid (SAHA) on cancer cell invasiveness and as a sensitizer for radio/chemotherapy in invasive cancer cells.

## RESULTS

### Differential expression of invasion-related genes and epithelial-mesenchymal transition (EMT) regulators in H-INV and L-INV cells

**Figure 1:**
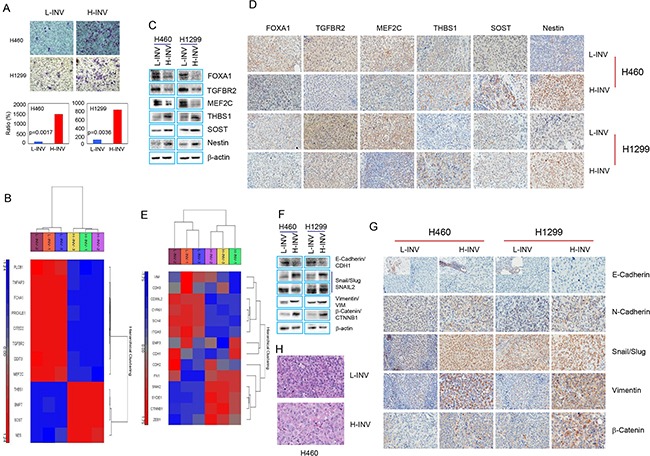
Differential expression of invasion-related genes and EMT regulators in H-INV and L-INV cells **A.** Invasion assay showing the differential invasiveness. Top: representative images of transwell invasion. Bottom: ratios of cell invasiveness (normalized to the percentage of invaded L-INV cells). p values were determined from three independent experiments. Error bars indicate standard deviation; **B.** Functional clustering of invasion-related genes in H460 H-INV versus H460 L-INV cells; **C.** Western blots showing the basal protein levels of invasion-related genes in cells; **D.** Representative IHC images of invasion-related proteins in xenograft tumors. Small images insets depict positive staining of E-cadherin found in edges of tumor specimen; **E.** Functional clustering analysis of EMT regulatory genes in H460 H-INV versus H460 L-INV cells; **F.** Western blots showing the basal protein levels of EMT regulators in cells. b-actin was included as loading control; **G.** Representative IHC images of EMT regulatory proteins in xenograft tumors; **H.** Representative images of H.E staining for xenograft tumors derived from H460 L-INV and H460 H-INV cells.

We determined the gene expression profile of H460 H-INV cells (versus H460 L-INV cells), using total RNA isolated from these cell subpopulations with validated differential invasiveness (Figure 1A). The transcriptional profile of H-INV was compared to that of L-INV (n=3), and 968 genes were found to be differentially expressed, with fold changes >2 and FDR <0.05. Figure 1B shows the functional clustering of invasion-related genes [9–14] that are differentially expressed in these two subpopulations. Using western blot analysis and immunohistochemical (IHC) staining, we validated the substantially lower protein expression of MEF2C, TGFBR2 and FOXA1, and higher protein levels of THBS1, SOST and nestin, in H460 H-INV cells and in H460 H-INV-derived xenograft tumors, when compared to that of H460 L-INV cells (Figure 1C and 1D). Similar results for differential protein expression were also observed for H1299 H-INV cells and its xenograft tumors, except for SOST which showed no difference for protein expression. We further noticed that the differential expression of MEF2C and TGFBR2 in xenograft tumors correlated with changes in protein expression in overall tumor cells, and the difference for expression of THBS1, FOXA1 and nestin resulted from the changes in the cell percentages with positive staining for the target proteins.

EMT is a process by which epithelial cells lose their cell polarity and cell-cell adhesion, and gain migratory and invasive properties to become mesenchymal stem cells. EMT is also a key step in the progression of tumors toward invasion and metastasis [15]. We therefore analyzed the gene profiles and protein expression of EMT regulators in the H-INV cells. Functional clustering analysis showed differential expression of several EMT regulators [16] in H460 H-INV cells versus H460 L-INV cells (Figure 1E). We further detected higher protein levels of N-cadherin, SNAIL/SLUG, and beta-catenin proteins in H-INV cells and in H-INV-derived xenograft tumors for both H460 and H1299 cell lines. Although gene profiling showed relatively lower mRNA expression for vimentin in H460 H-INV cells versus H460 L-INV cells, we detected higher vimentin protein expressions in the H-INV cells and in H-INV-derived xenograft tumors for both H460 and H1299 cell lines. In addition, western blot analysis showed lower E-cadherin expression levels in H460 H-INV and H1299 H-INV cells; however, we could not validate this in xenograft tumors because we did not detect positive staining for E-cadherin in the main sections of the specimen using four different commercially available antibodies. Of note, we observed some cells stained with E-cadherin in the edges of the tumor specimens and these staining showed no expression difference between H460 H-INV and H460 L-INV, and lower expression in H1299 H-INV-derived xenograft tumors when compared to that of H1299 L-INV (Figure 1F and 1G).

It is needed to be indicated that, we observed identical pathology for H460 H-INV-derived xenograft tumors versus H460 L-INV-derived xenograft tumors, and the former showing the tumor cells with larger nuclear. However, we also noticed that xenograft tumors derived from H1299 H-INV and H1299 L-INV cells showed similar pathology (Figure 1H and Supplementary Figure S1B).

### Enrichment of cancer stem cells (CSCs) in invasive H-INV cells

CSCs represent a subpopulation of tumor cells endowed with self-renewal and multi-lineage differentiation capacity, with the potential to give rise to differentiated progenies that can adapt to multiple target organ microenvironments and thus play essential roles in the metastatic spread of primary tumors [17, 18].

**Figure 2:**
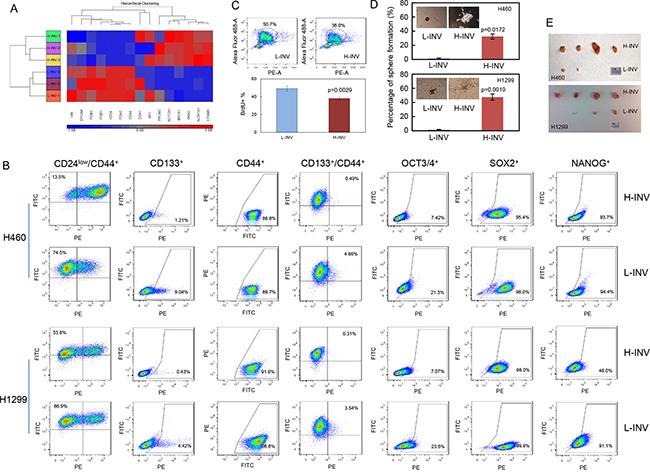
Enrichment of CSCs in H-INV cells **A.** Functional clustering of putative stem cell marker genes in H460 H-INV versus H460 L-INV cells; **B.** Flow cytometry analyses for putative CSCs markers; **C.** Graphs showing the difference of sphere numbers counted in sphere formation assay. The figure insets depict representative spheres; **D.** Tumorigenicity. Images showing the collected tumors from tumor initiating test; **E.** BrdU incorporation assay in H460 H-INV and H460 L-INV cells. Numbers indicate the percentage of BrdU-positive cells. p values were determined from three independent experiments. Error bar indicates standard deviation.

Functional clustering analysis showed that H460 H-INV cells express higher mRNA levels of the putative stem cell markers CD133 (PROM2) [19], ALDH1 [20] and BRCA1 [21], and the multiple pluripotent stem cell marker SOXII (SRY) [22], when compared to H460 L-INV cells (Figure 2A). H460 H-INV cells also express lower mRNA levels of CD24, which is proposed to be one of the molecular features when combined with CD44 expression (as CD44^+^/CD24^low^) for breast cancer stem cells [23]. In H-INV cells from both H460 and H1299 cell lines, we detected significant increases in the cell populations with positive staining of CD133, the combined marker CD44^+^/CD24^low^ and CD133^+^/CD44^+^, when compared to corresponding L-INV cells. We also detected increased cell percentages of OCT3/4-positive cells (Figure 2B and Supplementary Figure S1C). However, no difference was observed for the percentage of cells with positive staining of SOX II between the cells of H-INV and L-INV for both H460 and H1299 cell lines (Figure 2B). Of note, however, we observed dramatic shifts in the cell populations toward positive staining for SOX II in the H-INV cells (Supplementary Figure S1D), indicating increased SOX II expression in H-INV cells versus L-INV cells.

In addition, we detected a lower BrdU incorporation rate (49.4±3.10% for L-INV vs 38.1±1.91% for H-INV) in H460 H-INV cells, indicating that the H-INV cells are quiescent or slow cycling, a characteristic of CSCs [24] (Figure 2C).

We further performed sphere formation assays and tumorigenesis tests. Our results showed that, when compared to L-INV cells, H-INV cells from both H460 and H1299 cell lines produced increased numbers and sizes of pulmospheres in the semisolid matrix. We also observed different sphere structures formed with L-INV and H-INV cells, the latter showing an open structure with lineage-like spreading which may indicate the differentiation potential of the cells (Figure 2D). In tumorigenesis test, we found that all sites inoculated with H-INV cells (with 500 cells injection/mouse) developed tumors (4/4) by day 24 for both H460 and H1299 cell lines, with an average volume of 238 mm^3^ for H460 H-INV and 366 mm^3^ for H1299 H-INV cells (Supplementary Figure S1). On the other hand, two of four sites inoculated with same number of cells showed detectable tumors with an average volume of 5 mm^3^ for H460 L-INV, and three of four sites showed tumors with an average volume of 45 mm^3^ for H1299 L-INV (Figure 2E and Supplementary Figure S1). Most importantly, H-INV cells showed short latency for forming tumors when compared to L-INV cells (11±2 days versus 19±3 days for H460, and 10±3 days versus 18±2 days for H1299), and this, together with the BrdU assay results (Figure 2C), indicated a potential of that H-INV cells/xenograft grows at slower rate when compared to that of L-INV.

### Resistance of invasive H-INV cells to treatments with IR and chemotherapeutic agents

**Figure 3:**
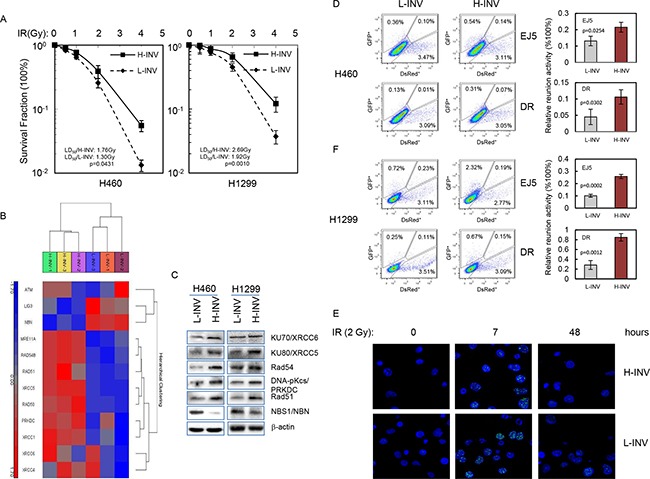
Resistance of H-INV cells to radiation treatment **A.** Clonogenic survival analysis showing the resistance of H-INV cells to IR treatment; **B.** Functional clustering of DNA damage repair genes in H460 H-INV versus H460 L-INV cells; **C.** Western blots showing the basal protein levels of DNA damage repair genes in cells. b-actin was included as loading control; **D.** *in vivo* reunion assays. Left: representative result of reunion analysis; right: Graphs showing the changes of relative HDR and NHEJ activity; **E.** Representative images of nuclear γ-H2A.X foci in irradiated H460 H-INV and H460 L-INV cells. Average LD_50_ and p values were determined from at least three independent experiments. Error bars indicate standard deviation.

We assessed the responses of H-INV and L-INV cells to IR treatment. Clonogenic survival analysis showed that both H460 H-INV and H1299 H-INV cells were more resistant to IR, when compared to corresponding L-INV cells, with dramatically increased survival fractions and LD_50_ values (Figure 3A).

The predominant mechanism by which therapeutic irradiation kills most tumor cells is through clonogenic death. In the process, DSBs are regarded as the specific lesions that initiate this lethal response [25], and the repair of DSBs is therefore critical in determining radiosensitivity [26]. Functional clustering showed that H460 H-INV cells expresses higher mRNA levels of DSB repair-relative genes such as DNA-PKcs, Ku80 and Rad51, when compared to H460 L-INV cells. We also detected higher protein levels of these genes in H-INV cells for H460 and H1299 cell lines (Figure 3B and 3C). These molecular features indicate that H-INV cells are with enhanced DNA damage repair capability. In support of this, we detected significantly higher *in vivo* reunion frequencies of NHEJ and HDR activity in H-INV cells (Figure 3D). We also observed relative persistence of γ-H2A.X nuclear foci, an indicator of lethal DNA damage with non-repaired DNA DSBs [27], in the H460 H-INV cells after IR treatment, when compared to the H460 L-INV cells (Figure 3E and Supplementary Figure S2).

**Figure 4:**
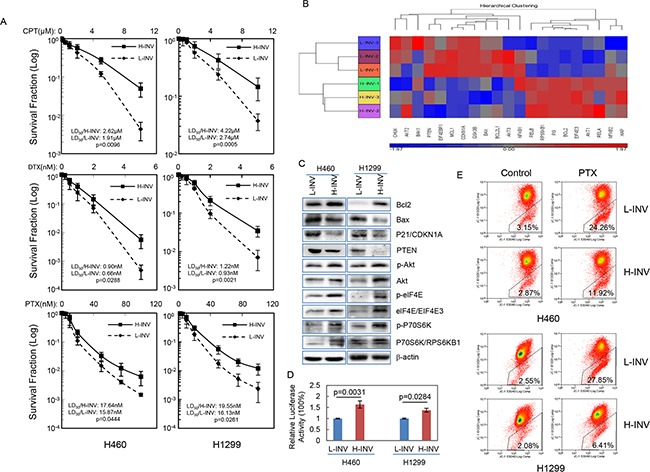
Resistance of H-INV cells to chemotherapeutic agents **A.** Clonogenic survival analyses showing the resistance of H-INV cells to treatment of chemotherapeutic agents; **B.** Functional clustering of cell survival-related genes in H460 H-INV versus H460 L-INV cells; **C.** Western blots showing the basal levels of protein and protein phosphorylation of survival-related genes in cells. b-actin was included as loading control; **D.** Relative NFkB activity; **E.** Mitochondrial apoptosis measured in cells treated with paclitaxel (PTX). Average LD_50_ and p values were determined from at least three independent experiments. Error bars indicate standard deviation.

Our results also showed that both H460 H-INV and H1299 H-INV cells are more resistant than the corresponding L-INV cells to treatments of cisplatin, docetaxel and paclitaxel (Figure 4A). Of interest, functional clustering analysis showed that genes correlated with activation of the PI3K, mTOR and NFkB pathways, as well as inhibition of mitochondrial apoptosis signaling, show increased expression in H460 H-INV cells versus H460 L-INV cells (Figure 4B). In H-INV cells isolated from both H460 and H1299 cell lines, we detected higher protein/phosphorylation levels of Akt/phospho-Akt (PI3K pathway) [28], elF4E/phospho-elF4E and P70S6K/phosphor-P70S6K (mTOR pathway) [29], higher protein levels of Bcl-2 (mitochondrial apoptosis pathway) [30] and lower protein levels of Bax, p21 and PTEN (Figure 4C). Using a luciferase reporter assay, we detected higher NFkB activity in H460 H-INV cells versus H460 L-INV cells (Figure 4D). These molecular events suggest that invasive lung cancer cells have the intrinsic properties of enhanced cell survival. Indeed, we detected less mitochondrial apoptosis in H460 H-INV and H1299 H-INV cells (versus that of L-INV cells) when cells were treated with paclitaxel (Figure 4E and Supplementary Data S2).

### Therapeutic potential of SAHA on invasive lung cancer cells

Our above results indicated that invasive human lung cancer cells, as a special subpopulation, show molecular signatures of cell invasion, EMT, DNA damage repair and cell survival signaling. These epigenetic characters not only reflect the heterogeneity of tumor nature but also indicate a potential of epigenetic changes leading to cancer cell invasion during tumor progress. Thus, it raises a possibility of epigenetic therapy for lung cancer invasion. We therefore investigated the effects of SAHA, an HDAC inhibitor that has shown promise in clinical trials as an epigenetic therapy for human malignancies [31], on H-INV cells.

**Figure 5:**
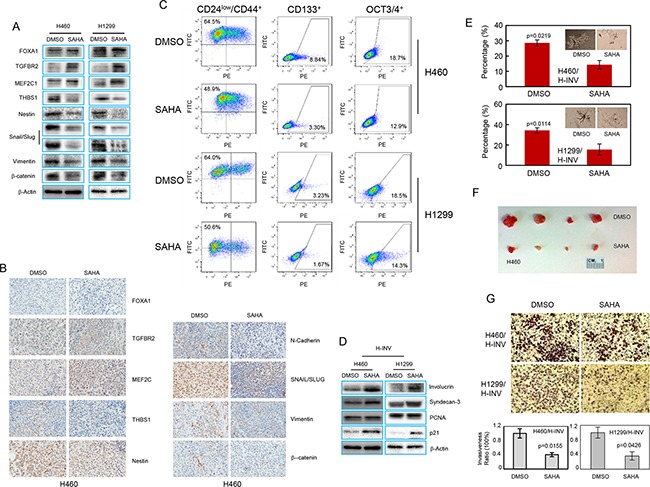
SAHA induces epigenetic modification and CSC differentiation in H-INV cells **A.** Western blots showing the effects of SAHA treatment on expressions of invasion-related and EMT regulator proteins in H-INV cells; **B.** Representative IHC images for invasion-related proteins and EMT regulators in xenograft tumors formed with H-INV cells with or without SAHA treatment; **C.** Flow cytometry analyses for putative CSCs markers; **D.** Western blot analyses showing the effects of SAHA treatment on expression of differentiation markers in H-INV cells. b-actin was included as loading control; **E.** Sphere formation assay showing the effects of SAHA treatment on sphere formation for H-INV cells. The figure insets depict representative spheres; **F.** Tumorigenicity of H-INV cells after SAHA treatment. Images showing the collected tumors from tumor initiating test; **G.** Cell invasion assay. Top: representative images of transwell invasion. Bottom: ratios of cell invasiveness. p values were determined from at least three independent experiments. Error bars indicate standard deviation.

We first determined the effects of SAHA on the expression of invasion-related and EMT-related genes. We found that treatment with 1 μM of SAHA for 72 hours increased the protein levels of TGFBR2 and MEF2C, and reduced the levels of THB1, Nestin, SNAIL/SLUG, Vimentin and b-catenin in H-INV subpopulations isolated from both H460 and H1299 cell lines (Figure 5A). We also detected increased protein levels for FOXA1 in SAHA-treated H1299 H-INV cells. However, no such changes could be observed for H460 H-INV cells (Figure 5A). In xenograft tumors formed with SAHA-treated H460 H-INV cells, we detected increased density of staining for TGFBR2 and MEF2C in overall tumor cells. In particular, we observed that SAHA treatment could significantly reduce the percentages of tumor cells with positive staining of THBS1, Nestin, N-cadherin, SNAIL/SLUG, Vimentin and b-catenin. We further noticed that, although the FOXA1 protein was barely detectable in control H460 H-INV-formed xenograft tumors, a few tumor cells showed positive staining for FOXA1 in xenograft tumors for SAHA-treated H460 H-INV cells (Figure 5B).

We next tested the effects of SAHA on enriched CSCs in H-INV subpopulation. As shown in Figure 5C, exposure to 1 μM SAHA for 72 hours significantly reduced the fraction of H460 H-INV and H1299 H-INV cells with positive staining of CD24^low^/CD44^+^ (from 62.3±2.37 to 52.0±2.87 for H460, and 61.0±3.75 to 52.3±1.54 for H1299), CD133 (from 7.39 ± 1.37 to 3.97 ± 0.72 for H460, and 3.04 ± 0.21 to 1.89 ± 0.19 for H1299) and OCT3/4 (form 19.57 ± 0.78 to 17.83 ± 0.25 for H460, and 18.07 ± 0.45 to 14.97 ± 0.59 for H1299) (Figure 5C and Supplementary Figure S3). We also detected increased protein expression of the differentiation markers involucrin and syndecan-3 [32, 33], as well as p21^Waf1/Cip1^, which served as a positive control for SAHA treatment [34], in H-INV cells after SAHA treatment (Figure 5D). We further noticed that SAHA treatment not only decreased the number of pulmospheres, but also led to the death, or at least inhibition of cell growth and expansion, of spheres formed with H-INV cells. However, tumorigenesis analysis showed that both control and SAHA-treated H460 H-INV cells formed tumors at all sites (4/4) in NOD/SCID mice by day 21. However, SAHA-treated cells showed a longer latency to tumor formation, when compared to control cells (10±4 days versus 18±2 days). The SAHA-treated H-INV cell tumors also showed an average volume of 49.8 mm^3^, which was significantly smaller than tumors derived from control cells (255.7 mm^3^) (Figure 5F and Supplementary Figure S3).

In addition, we observed reduced cancer cell invasiveness of H-INV cells when H-INV cells were treated with SAHA (Figure 5G).

**Figure 6:**
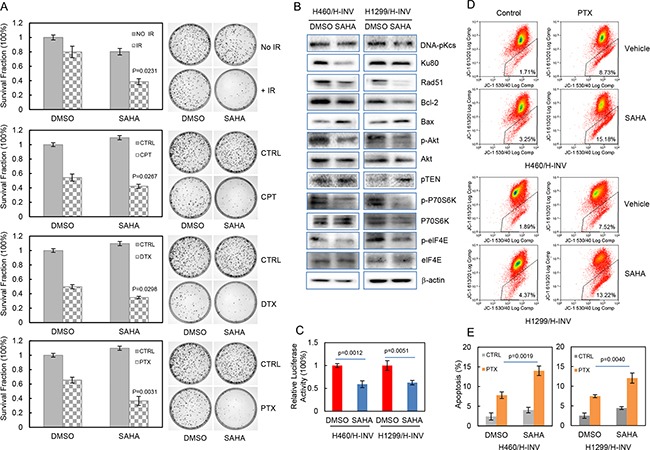
SAHA increases the sensitivity of H-INV cells to radiation and chemotherapeutic treatments **A.** Effects of SAHA on clonogenic survival of H-INV cells treated with IR and chemotherapeutic agents. Representative images showing the surviving colonies; **B.** Western blots showing the SAHA-induced changes in expression and phosphorylation of cell survival-related proteins in H-INV cells. b-actin was included as loading control; **C.** SAHA-induced changes of NFkB activity in H-INV cells; **D.** SAHA-induced changes of mitochondrial apoptosis in H-INV cells. Graphs showing the percentage of apoptosis. p values were determined from at least three independent experiments. Error bars indicate standard deviation. CPT = cisplatin, DTX = docetaxel, PTX = paclitaxel.

We further evaluated the potential effects of SAHA on the sensitivity of H460 H-INV cells to treatments of IR or chemotherapeutic agents, and the results showed that pretreatment with SAHA significantly reduced clonogenic survival of H460 H-INV cells in response to Cisplatin (2 μM), Paclitaxel (10 nM), Docetaxel (0.5 nM) or IR (2 Gy) (Figure 6A). SAHA treatment also caused obvious changes of protein expression for cell survival-related genes in H-INV cells of H460 and H1299 cell lines. These changes included down-regulation of Rad51, Ku80 and Bcl-2, and upregulation of Bax and PTEN in H-INV cells for both H460 and H1299 cell lines. Although SAHA treatment only slightly reduced Akt protein level in H460 H-INV cells and P70S6K protein level in H1299 H-INV cells, and showed no effects on expression of elF4E protein in both H460 H-INV and H1299 H-INV cells, we observed significant decreases in the phosphorylation levels of Akt, P70S6K and elF4E in SAHA-treated H-INV cells for both H460 and H1299 cell lines (Figure 6B). SAHA treatment also decreased the basal level of NFkB activity in H-INV cells (Figure 6C). These results indicated that exposure to SAHA could reduce cell survival capability, which was further supported by our findings that SAHA treatment led to increased apoptotic responses when cells were treated with Paclitaxel (Figure 6D and 6E).

These results indicated that SAHA treatment can lead to epigenetic changes, induce differentiation of enriched CSCs and reduce the invasiveness of invasive lung cancer subpopulation cells. SAHA treatment also increases the therapeutic responses of cells to IR and chemotherapeutic agents. It is needed to be indicated that, however, we barely detected changes of percentage for CD133-positive cells in H460 L-INV subpopulation (Supplementary Figure S3). It thus indicates a potential of that SAHA may differentially affect the L-INV and H-INV populations.

## DISCUSSION

Currently, there are about 300 open clinical and preclinical trials (www.ClinicalTrials.gov) focusing on the development of new diagnostic and therapeutic strategies for lung cancer metastases. It not only illustrates the critical need for scientific research, but also indicates the complicated and multivariable nature of lung cancer metastasis, and a comprehensive knowledge of the metastatic process is leading to improved cancer treatment.

We showed in this study that invasive cancer cell subpopulations of H460 and H1299 cells show positive molecular signatures for not only cell invasion, but also EMT and CSCs. EMT is a cell-biological program that dissociates the cells within epithelial cell sheets into individual cells that exhibit multiple mesenchymal attributes, and allows epithelial cells acquire the characteristics of invasive mesenchymal cells [35, 36]. Re-initiation of the EMT program has been believed to play a critical role in promoting cancer cell invasion [37]. CSCs, as a special subgroup of cells that exist in heterogeneous primary tumors and are with the abilities to form early micrometastases and survive after chemotherapy thus correlated to recurrence of cancer at distal sites in patients [6], have been identified in many types of cancer including NSCLC [6, 24, 38, 39]. Our results showed that CSC-enriched invasive H-INV cells were more resistant to treatments of IR and chemotherapeutic agents, and these biological responses may correlate to the observed molecular events of elevated DNA damage repair and cell survival signaling. Of interest, we detected lower expression of PTEN in invasive H-INV cells. Recent study has shown that PTEN loss induces EMT in human cancer cells [40]. These findings further support our conclusion that human lung cancer invasive cells, as a subset cancer cell population, display epigenetic features that allow cell invasion and survival.

Epigenetic regulation is a dynamic process, and the most diverse epigenetic modifications occur on histone proteins [41, 42]. Post-translational modifications that occur on certain amino acid residues of histone protein tails modify chromatin structure and alter gene expression. Among the covalent histone modifications, histone acetylation is the most understood. The HDAC enzymes, together with their counterparts the histone acetyl-transferases (HATs), control the level of histone acetylation. Studies have demonstrated that HDAC inhibitors (HDACis) induce tumor cell apoptosis, growth arrest, senescence, differentiation, and immunogenicity, and inhibit angiogenesis. HDACis are therefore of great interest for cancer epigenetic therapy. In this study, we demonstrated the epigenetic modifications and therapeutic effects of SAHA (vorinostat), an HDACi approved for clinical trials for treatment of lung cancer [43], on invasive lung cancer subpopulation cells, suggesting a clinic potential of SAHA for NSCLC patients. Of note, these effects of SAHA on invasive cancer cells are observed not only in H460 cells that carries wild-type p53 protein, but also in H1299 cells which expresses no p53. Mutation of the p53 gene is one of the most significant molecular events occurring in about 50% of NSCLC and correlate with the resistance to clinical treatments [44]. Our results thus suggest the therapeutic potential of SAHA may benefit overall NSCLC patients. However, future studies are needed to evaluate the clinical applicability of SAHA as a part of the chemotherapeutic regimen for NSCLC.

In addition, we noticed higher expression of THBS1 in H-INV cells and in H-INV-derived xenograft tumors, and SAHA treatment reduced THBS1 expression. THBS1 has been shown to act as a tumor suppressor in lung cancer, and reduced expression of THBS1 indicate poor prognosis in NSCLC patients [45, 46]. However, other studies have revealed that THBS1 promotes EMT transition and cancer cell invasion [13, 47]. These results suggest diversity of function for THBS1, and the role of THBS1 in cancer metastasis of NSCLC thus needs further investigation.

## MATERIALS AND METHODS

### Cell line and reagents

H460 and H1299 cell lines were obtained from ATCC, which has provided certifications (see DDCM752847-1007392-40291-H1299.pdf and DDCM752847-1007393-99465-H460.pdf in supplementary materials) of analysis for karyotyping and short tandem repeat (STR) profiling. The cells were grown in RPMI-1640 medium supplemented with 10% fetal bovine serum (FBS) (Gemini). Both cell lines were tested negative for mycoplasma contamination using a Cell Culture Contamination Detection Kit (ThermoFisher Scientific).

Docetaxel, Paclitaxel, Cisplatin, Gemcitabine and SAHA were purchased from Sigma-Aldrich. The antibodies were from Abcam (PTEN, DNA-PKcs, Ku80, SNAIL+SLUG and Vimentin), Cell Signaling Technology (phospho-histone H2A.1 [Ser-139], phosphor-Akt1 [Ser-473], Akt1, phosphor-p70 S6 Kinase [Thr-389], p70 S6 Kinase, phosphor-elF4E [Ser-209], elF4E, Ku70, NBS1 and β-actin), Santa Cruz Biotechnology (p21, p53, Bcl-2, BAX, PCNA, RAD51, RAD54, Ki67, syndecan-3 and involucrin) and One World Lab (MEF2C, SOST, Thrombospondin I, Nestin, FOXA-1, TGFBR2, beta-catenin, E-cadherin and N-cadherin). The plasmids pimEJ5GFP (http://www.addgene.org/44026), pDRGFP (http://www.addgene.org/26475) and pDsReD-Express2-N1 (Clontech) were provided by Dr. Jeremy Stark (City of Hope). Enzyme I-SceI was from New England Biolabs.

### Collection of cancer cell subpopulations with differential invasiveness

Cell subpopulations with differential invasiveness were collected from H460 and H1299 cell lines with using Boyden Chamber-based isolation as described in details in Supplementary Methods. Briefly, cells were suspended in growth medium containing 10% FBS and were seeded in 1× BME (Trevigen)-coated 8.0 μm pore size Boyden-type cell culture inserts (Millipore). 72 hours later, the cells remaining in the inserts were collected as the cell population with low invasiveness (L-INV), and invading cells that migrated into the bottom vessels were collected as the cell population with high invasiveness (H-INV). The collected cells were then maintained in glucose-free RPMI-1640 medium with 10% FBS. An invasion assay was conducted to validate the differential invasiveness of these cell populations when the cells were used for the experiments in this study.

### Invasion assay

5×10^4^ cells in growth medium containing 1% FBS were seeded in 1× BME-coated cell culture inserts. Complete growth medium containing 10% FBS was placed outside the chambers, and the cells were allowed to invade toward the attractant of 10% FBS medium. Twenty-four hours later, invasive cells were visualized and counted as previously described [48].

### Microarray experiment

Total RNA was prepared from three different sets of H460 H-INV and H460 L-INV cells using Trizol reagent (Thermo Fisher). Illumina TotalPrep RNA Amplification Kit (Thermo Fisher) was used to transcribe 100 ng total RNA to cRNA, according to the manufacturer's instructions. A total of 750 ng of cRNA was hybridized at 58°C for 16 hours to the Illumina Human HT-12 v4 Expression BeadChips. The BeadChips were scanned using HiScan Software on the HiScan system (Illumina).

### Microarray data analysis

The raw data files were processed using GenomeStudio software (Illumina) with background correction and quantity normalization, and further imported to Partek Genomic Suite 6.6 (Partek) with log transformation for data analysis. One-way ANOVA analysis was used to identify lists of differentially expressed genes. The data were filtered using criteria of fold change > 2 and p value with Benjamini-Hochberg false discovery rate (FDR) step up < 0.05. Differentially expressed genes were subjected to hierarchical clustering using the Partek Genomics Suite and to functional clustering analysis using the web-based DAVID Bioinformatics Resources 6.7 (http://david.abcc.ncifcrf.gov/summary.jsp) [49, 50].

The data discussed in this publication have been deposited in the Gene Expression Omnibus (NCBI, accession number GSE68916).

### NF-κB luciferase assay

Luciferase reporters containing specific consensus sequences for NF-κB and β-gal (as a control) were transfected into cells using electroporation (Gene Pulse Xcell, Bio-Rad), and luciferase activity was measured and normalized to b-gal activity as transcriptional activity of NF-κB, according to the manufacturer's direction (Promega).

### Flow cytometric analysis

2x10^6^ cells were collected and stained with 20 μ of phycoerythrin (PE)-conjugated anti-CD24, anti-Sox2, anti-Oct3/4, and anti-Nanog antibodies, or fluorescein isothiocyanate (FITC)-conjugated anti-CD44 (BD Biosciences), or with PE-conjugated anti-CD133 (Miltenyi Biotech), or co-stained with FITC-conjugated anti-CD44 antibodies and PE-conjugated anti-CD24 or PE-conjugated anti-CD133. In the process of staining for Sox 2, Oct3/4 and Nanog, BD Perm/Wash buffer (BD biosciences) was used according to the manufacturer's instructions. PE- or FITC-positive cells were quantified on a LSRII flow cytometer (BD Biosciences), and up to 5x10^4^ cells were counted per run.

For the bromodeoxyuridine (BrdU) incorporation assay, 10 μM BrdU was added to the cell suspension 2 hours before collection. Cells were then fixed with cold 70% ethanol, and labeled with a FITC-conjugated anti-BrdU monoclonal antibody according to the manufacturer's instructions (BD Biosciences). Propidium iodide was added before flow cytometric analysis. Detection of BrdU incorporation in DNA synthesizing cells was conducted by flow cytometry.

### Clonogenic survival analysis

500-1000 cells were plated in 60-mm dish, and were treated with the indicated chemotherapeutic agents for 48 hours or with ionizing radiation (IR). DMSO was included as control. Cells were then washed twice with growth medium, and maintained in culture vessels for 10-14 days. After staining with crystal violet, colonies consisting of >50 cells were considered as surviving colonies and directly scored using an inverted microscope. Average numbers for surviving colonies were plotted versus control to determine the 50% lethal doses (LD_50_) for each treatment, or survival fractions. When SAHA was applied, cells were pretreated with 1 μM SAHA, or DMSO as a control, for 72 hours before plating.

### Western blot analysis

The cell lysates were prepared in RIPA buffer with mild sonication, and subjected to SDS-PAGE gel electrophoresis for the immunoblot assays. Membranes were striped with SDS solution, and were reprobed with b-actin for validation of equivalent protein loading.

### Assay of mitochondrial membrane potential (MMP)

Cells were treated for 72 hours with 10 nM of paclitaxel, and MMP was analyzed by JC-1 staining according to manufecture's instruction (MitoProbe JC-1 Assay kit, Life technologies). Briefly, 1×10^6^ cells were collected and suspended in fresh medium and stained with 2μM of JC-1 for 15 minutes. Fluorescence was monitored by using flow cytometry, measuring both the monomer (527-nm emission; green) and J-aggregate (590-nm emission; red) forms of JC-1 following 488-nm excitation. The percentage of monomeric form or fluorescence green was then quantified as the MMP.

### Non-homologous end-joining (NHEJ) and homology-directed repair (HDR) reunion assays

*In vitro* reunion assays were based on the reactivation of linearized plasmid as previously reported [51]. Briefly, 1x10^5^ cells were co-transfected with 1.2 μg I-SceI-linearized EJ5-GFP or DR-GFP substrates and 0.5 μg circular pDsReD-Express2-N1 (as a transfection control) using electroporation. The cells were then treated with 2 Gy of IR. Flow cytometry analysis was performed at 72 hours, and the ratio of GFP-positive cells to DsRed-positive cells was used as a measure of relative activity for EJ5 (NHEJ) or DR (HDR).

### Immunofluorescence analysis

After the treatments, cells were washed twice with PBS, and fixed in 4% paraformaldehyde. Immunofluorescence analysis for γ-H2A.X was performed as previously reported [51]. Images were acquired with a LSM 510 confocal microscope (Zeiss) with a 40X objective. At least 100 cells from each experiment were selected at random and were counted to calculate the percentage of cells as “positive” for γ-H2A.X foci if they displayed >5 discrete dots in the nuclei.

### Sphere forming assay

Sphere-forming assay was performed as described previously [52] with modifications. Briefly, 100 freshly isolated cells were gently mixed with 100 μL of Matrigel Basement Membrane Matrix (BD Biosciences), and were added into a 24-well plate. The plate was left upside down at room temperature for 5 minutes, and the cells were then cultured under serum-free conditions with supplements of 20 ng/ml of epidermal growth factor (EGF), 10 ng/ml of basic fibroblast growth factor (bFGF) and B27 supplement (Life Technologies). Sphere formation was evaluated at day 18 using microscopic imaging.

### Xenograft and tumor initiating test

The animal protocol (Project # SCXK2008-0016) was approved by the Institute Animal Ethical Committee at Zhejiang Academy of Medical Sciences (Hangzhou, China).

For xenograft, 1×10^6^ H-INV and L-INV cells were suspended in 0.2 ml of HBSS/Matrigel (Life technologies) mixture (1:1 V/V), and were inoculated subcutaneously (s.c.) into the bilateral flanks of same animal of six-week-old female NOD/SCID mice (Charles River, Beijing). The formed tumors were collected 10 days after injection and fixed in formalin solution.

Tumor initiating test was conducted following previously reported methods [53]. Briefly, H-INV and L-INV cells were resuspended in serum-free PBS/Matrigel mixture (1:1 V/V), and 500 cells were inoculated s.c. into the bilateral flanks of the same animal (Charles River, China). The mice were euthanized three to four weeks later. The tumor volumes were determined from caliper measurements of tumor length (L) and width (W), according to the formula (LxW2)/2, and hematoxylin and eosin (H&E) staining was performed for validation of tumors formed.

For SAHA application, cells were treated with 1 μM SAHA, or DMSO as a control, for 72 hours before inoculation. SAHA (20 mg/kg/day) was delivered for five days after inoculation of the cancer cells by intraperitoneal injection.

### Immunohistochemistry

Xenograft tumor sections were deparaffinized and rehydrated. Endogenous peroxidase activity was blocked with 3% hydrogen peroxide in methanol. Heat-induced antigen retrieval (HIER) was carried out for all sections in 0.01 M citrate buffer, pH 6.0, using a steamer at 95°C. All primary antibodies were diluted with BSA to a concentration of 1:50 and applied to the sections. Incubation was for 45 minutes at room temperature followed by incubation with a Dako EnVision+System-HRP Labelled Polymer for 30 min at room temperature. Diaminobenzidine was then applied for 10 min. The sections were counterstained with hematoxylin, dehydrated, coverslipped and visualized.

### Statistical analyses

Statistical analyses were performed using Student's t-test. A p value <0.05 was considered significant (*).

## SUPPLEMENTAL DATA






