# Supplementary material for: Epigenetic therapy potential of suberoylanilide hydroxamic acid on invasive human non-small cell lung cancer cells
Source: Oncotarget. 2016 Sep 12;7(42):68768–80. doi: 10.18632/oncotarget.11967 (PMC5356588; doi:10.18632/oncotarget.11967)
Supplement: Supplementary file 1 [file oncotarget-07-68768-s001.pdf]

Epigenetic therapy potential of suberoylanilide hydroxamic acid on invasive human non-small cell lung cancer cells

SUPPLEMENTAL DATA

SUPPLEMENTARY METHODS

Collection and maintenance of cancer cell populations with differential invasiveness

H460 or H1299 cells in growth medium containing 10% FBS were seeded in multiple 8.0-µm pore size cell culture inserts (1×10^6/insert) of Boyden-type cell invasion chambers (for 6-well plate, Millipore, Danvers, MA) coated with 1× BME (Trevigen, Gaithersburg, MD). Complete growth medium was also placed outside the chambers. 72 hours later, cells remained in top inserts were collected as cell population with low invasiveness, and cells invaded/migrated into the bottom vessels were collected as cell population with high invasiveness. Collected cells were maintained in Glucose-free RPMI-1640 medium with 10%FBS. After cells reach 70% confluences, cells were trypsinized and were maintained in Glucose-free RPMI-1640 medium for two passages. Cells were then collected and re-seeded in 1× BME coated cell culture inserts for next round of selection. In this second round of selection, cells remained in top inserts with plated cells that were previously collected from top insert in first round selection were collected as cell population with low invasiveness, and cells grown in bottom culture vessels with plated cells that were previously collected from bottom vessels in first round selection were collected as cell population with high invasiveness. After four rounds of selections, cells remained in top inserts were collected as low-invasive cells (L-INV), and cells grown in bottom culture vessels were collected as high-invasive cells (H-INV). We detected differential invasiveness with significance for these cells populations. According to our observation, the differential invasiveness for L-INV versus H-INV cells could last for up to five passages when cells were maintained in Glucose-free RPMI-1640 medium, and then H-INV cells were with gradually losing of the capability of high-invasiveness during the further passaging. We thus collected L-INV and H-INV cells that were passaged two times after the fourth selection, and stored these cells in liquid nitrogen as stocks. A diagram showing the isolation strategy of L-INV and H-INV cell populations was shown in Supplementary Figure S1A.

When cells were needed for the designed experiments, cells were thawed and grown in Glucose-free RPMI-1640 medium. Cells were then reselected for one more time with Boyden chamber-based isolation method as described above. The experiments with these cell populations were performed within two passages after thawing and re-selection.

The invasive assays were conducted to validate the differential invasiveness of these cell populations when cells were used for the designed experiments in this study.

RNA samples for microarray experiment

L-INV and H-INV cell populations of H460 were isolated as described above. After thawing the stocked cells, cells were reselected once with Boyden chamber-based isolation method and grown in Glucose-free RPMI-1640 medium. The differential invasiveness of these cell populations were validated with invasive assay. Total RNA was prepared from three different sets of H460 H-INV and H460 L-INV cells with using Trizol reagent.

SUPPLEMENTARY FIGURES

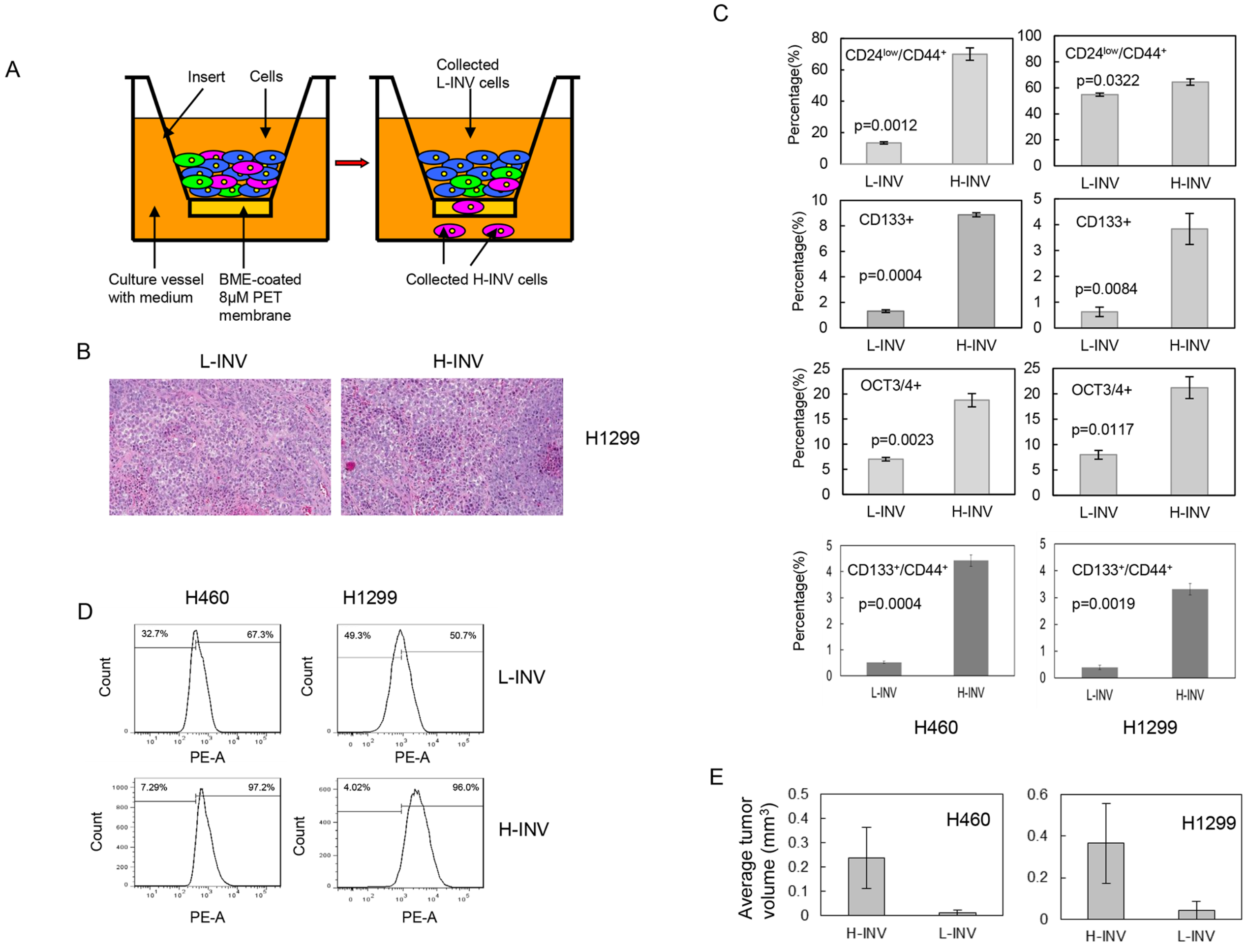

Supplementary Figure S1: A. Illustration of strategy for isolating cancer cell populations with high (H-INV) and low (L-INV) invasiveness; **B.** Representative images of H.E staining for xenograft tumors derived from H1299 L-INV and H1299 H-INV cells; **C.** Graphs showing the differences of the cell fractions with positive staining of stem cell markers $CD44^+/CD24^{low}$, $CD133^+$, $CD133^+/CD44^+$ and $OCT3/4^+$ in H-INV and L-INV cells, as shown in Figure 2B; **D.** Representative images showing the shift of SOXII-positive cell fractions in H-INV cells comparing to L-INV cells detected in flow cytometric analysis; **E.** Diagram showing the average of tumor volumes in tumor initiation tests, as shown in figure 2D, for H-INV and L-INV cells.

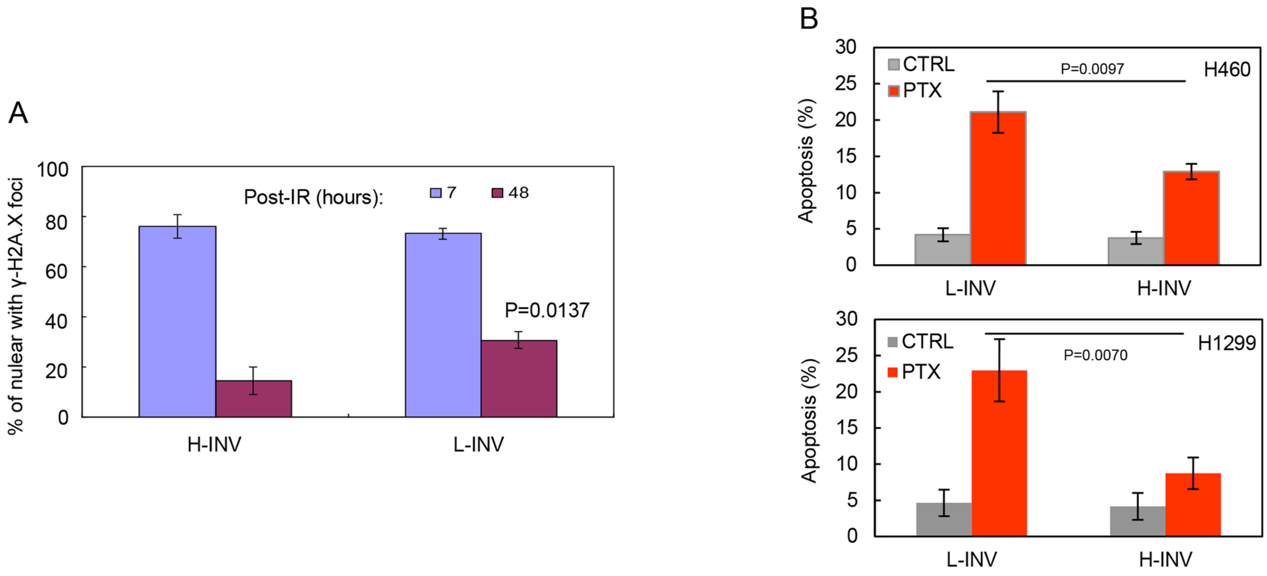

Supplementary Figure S2: A. Graph showing the significant difference on the kinetic persistence of γ-H2A.X foci, as shown in Figure 3E, in H460 H-INV and H460 L-INV cells in response to 2 Gy of IR; **B.** Graph showing the difference of mitochondrial apoptosis in paclitaxel-treated H-INV and L-INV cells, as shown in Figure 4E.

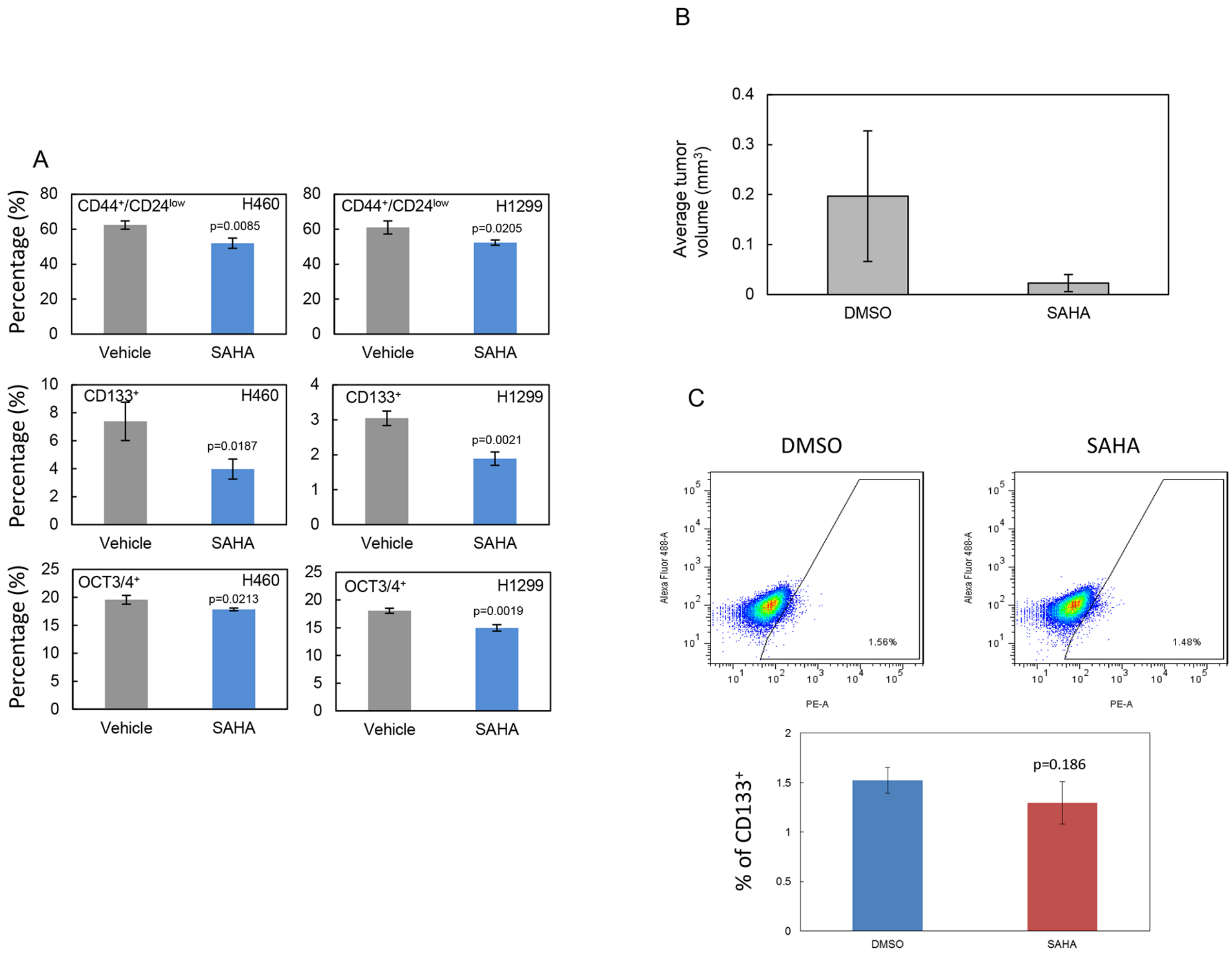

Supplementary Figure S3: A. Graphs showing the effects of SAHA treatment (1 μM for 72 hours) on cell fractions with positive staining of putative stem cell markers $CD44^{+}/CD24^{low}$, CD133 and OCT3/4 in H-INV cells, as shown in Figure 5C; **B.** Graph showing the effect of SAHA treatment on tumor size for tumors formed in tumoregenesity with H460 H-INV cells in NOD/SCID mouse, as shown in Figure 5F. **C.** Flow cytometry analysis for CD133 staining in H460 L-INV cells. Top: representative results for flow cytometric analysis; bottom: Graph showing the effect of SAHA treatment on changes of cell fraction with positive staining of CD133.
