# Supplementary material for: Epigenetic therapy potential of suberoylanilide hydroxamic acid on invasive human non-small cell lung cancer cells
Source: Oncotarget. 2016 Sep 12;7(42):68768–80. doi: 10.18632/oncotarget.11967 (PMC5356588; doi:10.18632/oncotarget.11967)

DDC MEDICAL

Certificate of Analysis

Cell Line DNA Typing Report

Case	DDCM752847
Sample Name	752847
Sample Type	DNA Card
Sample Number	AAC04319
Date Received	1/20/2016

Locus	Allele Sizes		Locus	Allele Sizes	
D3S1358	17		TH01	6	9.3
vWA	16	18	FGA	20	
D16S539	12	13	D5S818	11	
CSF1PO	12		D13S317	12	
TPOX	8		D7S820	10	
D8S1179	10	13	D2S1338	23	24
D21S11	32.2		Penta D	13	
D18S51	16		Penta E	11	
D19S433	14		Amelogenin	X	

RN: 1007392

Results indicate the allele(s) detected at each locus tested. Each allele represents the number of short tandem repeats present at that locus. Generally, a DNA profile uniquely identifies an individual cell line. However, some cell lines may exhibit genomic instability over time leading to slight changes in the DNA profile. Additional guidance about such changes and their significance can be found in the ASN-0002 document.

DDC is accredited/certified by CAP, ISO/IEC 17025:2005 through ACLASS

This test is accredited and meets the requirements of ISO/IEC 17025:2005 as verified by ACLASS. Refer to certificate and scope of accreditation AT-1299

Based on the samples received, I, the undersigned Laboratory Director, declare the genetic data is correct as reported on 1/20/2016.

Thomas M. Reid, Ph.D. | Laboratory Director

DDC Medical One DDC Way Fairfield OH Fairfield 1-888-362-3928

DNA DIAGNOSTICS CENTER · DDC MEDICAL · THE DNA EXPERTS

Allele Report

Sample 5: AAC04319_B07_031_0971.fsa Run date and time: 01/14/2016 - 18:34:08 -> 01/14/2016 - 19:05:29

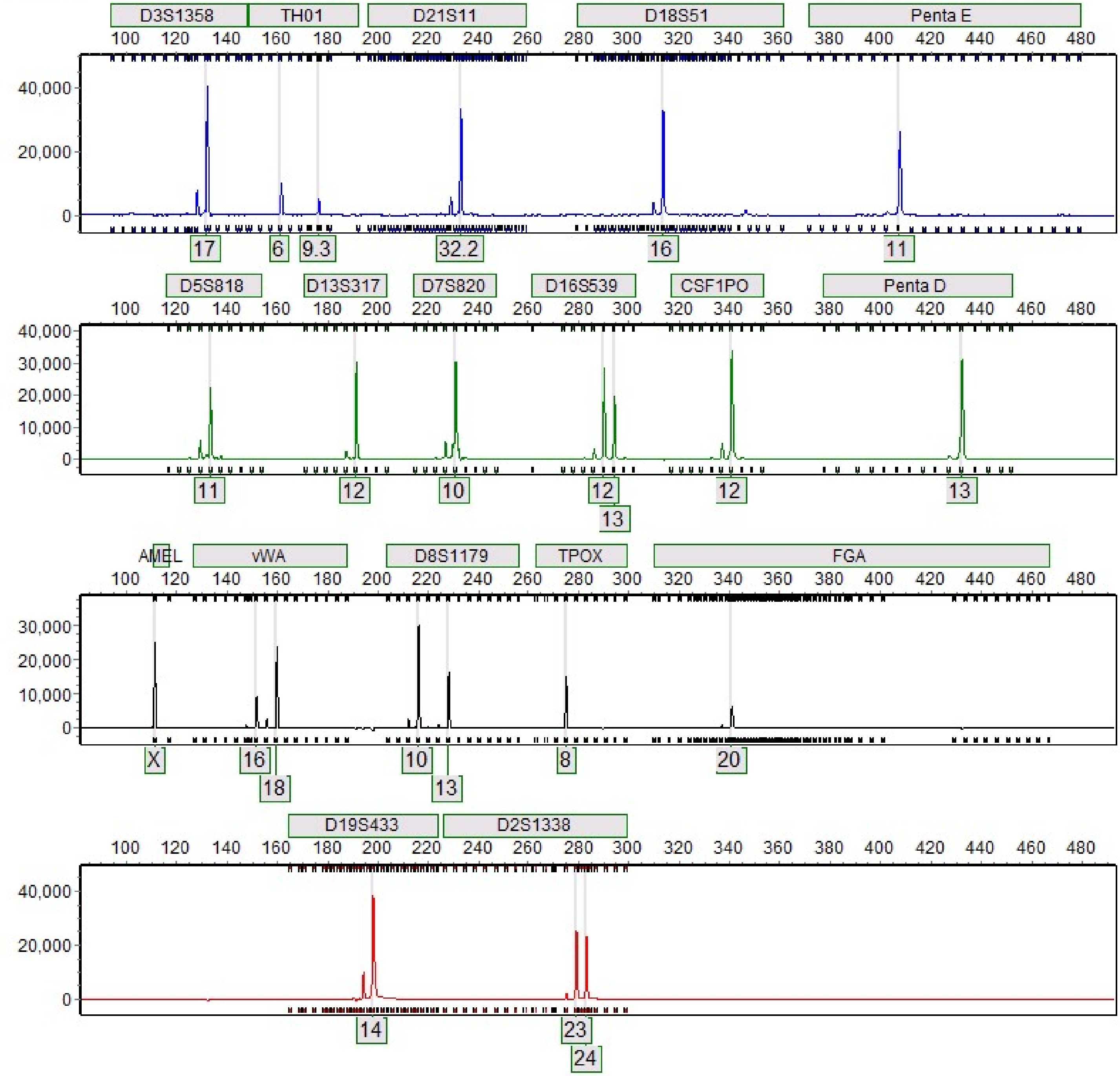

Supplement: Supplementary file 2 [file oncotarget-07-68768-s002.pdf]
